# Plyometric training increases thickness and volume of knee articular cartilage in mice

**DOI:** 10.1038/s41526-025-00458-z

**Published:** 2025-02-13

**Authors:** Marco Chiaberge, Neelima Thottappillil, Anna-Maria Liphardt, Anderson Furlanetto, Dylan Odell, Christine Wang, Stephen Hope, Stephen Smee, Joseph Rehfus, Anja Niehoff, Mark Shelhamer, Colin Norman, Marc J. Philippon, Johnny Huard, Aaron W. James, Chen-Ming Fan

**Affiliations:** 1 The William H. Miller III Department of Physics & Astronomy, Johns Hopkins University, Baltimore, MD USA; 2 Space Telescope Science Institute for the European Space Agency (ESA), ESA Office, 3700 San Martin Drive, Baltimore, MD USA; 3 Department of Pathology, Johns Hopkins University School of Medicine, Baltimore, MD USA; 4 Department of Internal Medicine 3–Rheumatology and Immunology, Universitätsklinikum Erlangen, Friedrich-Alexander-Universität (FAU) Erlangen-Nürnberg, Erlangen, Germany; 5 Deutsches Zentrum Immuntherapie, Universitätsklinikum Erlangen, FAU Erlangen-Nürnberg, Erlangen, Germany; 6 Department of Mechanical Engineering, Johns Hopkins University, Baltimore, MD USA; 7 Department of Neuroscience, Johns Hopkins University, Baltimore, MD USA; 8 Department of Biomedical Engineering, Johns Hopkins University, Baltimore, MD USA; 9 Department of Biology, Johns Hopkins University, Baltimore, MD 21218 USA; 10 Institute of Biomechanics and Orthopaedics, German Sport University Cologne, Köln, Germany; 11 Cologne Center for Musculoskeletal Biomechanics, University of Cologne, Faculty of Medicine, Köln, Germany; 12 Human Spaceflight Lab, Johns Hopkins School of Medicine, Baltimore, MD USA; 13 Steadman Philippon Research Institute, Vail, CO USA; 14 The Steadman Clinic, Vail, CO USA; 15 Department of Embryology, Carnegie Institution for Science, Baltimore, MD 21218 USA

**Keywords:** Physiology, Anatomy

## Abstract

Degeneration and thinning of articular cartilage lead to osteoarthritis and may result from reduced joint loading during e.g. bed rest or as a result of microgravity during space flight. Anabolic physical exercises for cartilage are not well studied to date. We built an experimental apparatus for plyometric training with mice to test potential benefits of jumping for articular cartilage. The exercise group (JUMP) performed jump training for 9 weeks and was compared with sedentary mice (control, CON) and hindlimb-suspended (HLS) mice (to simulate reduced loading) for the same duration. Knee cartilage was assessed via 3-dimensional reconstruction of micro-CT scans and histology. We observed significant thinning and volume reduction of articular cartilage at the medial tibial-femoral point of contact in the HLS group. Clustering of chondrocytes was present in HLS. By contrast, the JUMP group showed both cartilage thickening and volume increase. We observed a similar trend on trabecular bone thickness and volume. Our results show that plyometric training can stimulate cartilage thickness and volume in mice. This suggests further investigation of this mode of exercise as a countermeasure to prevent cartilage atrophy in disuse scenarios such as long duration spaceflight, and for patients at risk of developing osteoarthritis.

## Introduction

The microgravity environment during long duration space flight results in catabolic adaptations of the musculoskeletal system leading to reduced physical function and increased injury risk, especially upon return into higher gravitational loads on earth. The human musculoskeletal system has been studied extensively in this context with special attention devoted to skeletal muscles and bones. Exercise countermeasures for International Space Station (ISS) crews are continuously optimized to preserve tissue health and comprise both cardiovascular and strength training. To date, there is little research on the adaptation of articular cartilage to microgravity and current exercise countermeasures have not been designed with regards to cartilage health^1–3^.

Healthy articular cartilage is the prerequisite for unrestricted physical activity. In synovial joints the presence of synovial fluid and the lining of articular hyaline cartilage enables low friction during joint movements. Composition and morphology of articular cartilage is optimized for load-bearing function and allows to sustain repetitive, high mechanical loads occurring during all load bearing activities. Mechanical load is essential for cartilage health, and immobilization -because of illness, injury or bed rest- changes tissue biology and structure^2,4–8^. Simple locomotion replacement training -including squats, heel raises and a small number of hops-, and resistive vibration exercise alone or combined with protein and bicarbonate supplementation does not provide sufficient countermeasures for bed rest induced changes in cartilage biology^9,10^. There is also evidence that excessive loading caused by mechanical compression may result in tissue catabolism, damage of collagen and chondrocyte death^11–13^. In light of the above evidence, a preventative or rehabilitative exercise for cartilage health is highly desired^4,14^. But potential benefits, as opposed to any induced damage, must be carefully evaluated in a well-controlled environment to ensure that positive and negative effects of the activities are well understood.

Animal models have been used for many decades to investigate the adaptation of articular cartilage to changes in the mechanical environment. Rodent models allow us to investigate intervention effects in articular cartilage more quickly and in a more standardized manner than in humans. Previous studies^15–18^ investigated the effect of both joint unloading and radiation in rats and mice. Those animals were hindlimb unloaded via tail suspension for up to 2 months and signatures of osteoarthritis and significant changes in the structure of the hip and knee cartilage were seen^16^. These include clustering of chondrocytes and increased circulating serum cartilage oligomeric matrix protein (sCOMP), both indicators of cartilage damage. Mice showed gait changes and arthritic phenotypes after 35 days aboard the ISS^18^. In particular, hindlimb suspension^16^ and spaceflight^19^ selectively affects the tibial-femoral cartilage-cartilage point of contact. Cartilage damage appeared to be permanent, even after full weight bearing was resumed in a sample of mice that were both suspended and irradiated^17^. Yet, some level of recovery was seen with simple exercise such as walking or climbing, after ground based hindlimb suspension^17,19,20^. It has also been shown that treadmill running may mitigate cartilage degradation in HLS rats^21^. An important question is what other exercise regimen can prevent articular cartilage degradation, support its recovery or even improve cartilage health (e.g. thickness) in preparation for long duration spaceflight.

There is growing evidence for cartilage degradation in humans in response to prolonged spaceflight. Initial results of the ESA-Cartilage experiment suggest the initiation of cartilage degeneration during 4–6 months ISS mission^7^. This could pose a serious health problem for crew in longer duration missions, such as a mission to Mars, or for a long-term permanence on the Moon in the planned NASA Artemis lunar base. Because cartilage is a tissue with limited capacity for self-repair and regeneration^22^, the potential for pre- and inflight exercise routines to preserve cartilage health during periods of immobilization should be explored.

Space agencies support ISS crew with specific pre- and post-flight exercise programs and exercise routines while on board the ISS^8^. Pre-flight training prepares the astronauts to the challenges of space flight by strengthening the musculoskeletal system, and post-flight routines focus on comprehensive reconditioning. In-space routines are aimed at mitigating the deconditioning and damaging effects of microgravity using both strength and cardiovascular exercises. However, while more research is needed to further optimize the existing training protocols, specific strategies need to be developed as a countermeasure to joint cartilage degradation in space, which is still not considered as yet^1^.

Cyclic mechanical compression was shown to produce a positive effect on cartilage explants^23^. Plyometric training may be used to produce cyclic compression on joint cartilage. This mode of exercise is also known to enhance secretion of hormones such as growth hormone and insulin-like growth factor (IGF-1) in humans^24^. IGF-1 is a biochemical regulator of articular cartilage and it was shown to stimulate protein synthesis alone or in combination with low-frequency cyclic dynamic compression^25^. These clues together suggest a possible benefit of plyometric training to knee cartilage.

**Fig. 1:**
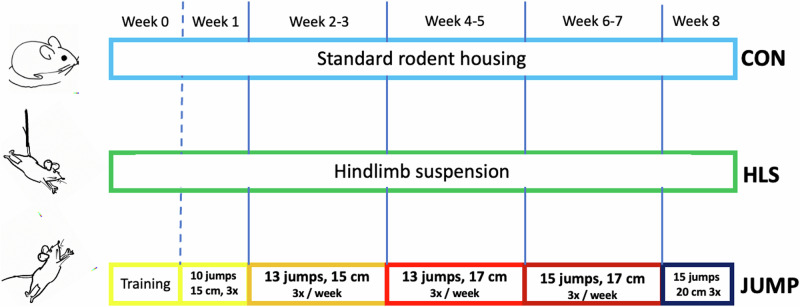
Schematic of experimental design. The timeline of the activity of the three mouse groups: control (CON), hindlimb suspended (HLS), and jumping (JUMP) is shown. CON and HLS groups were not subjected to any additional activity. The JUMP group exercised 3 times per week, performing the number of jumps and height indicated in the figure in each session.

Here we report results of an experiment showing that indeed plyometric (jump) training is an exercise that can be used to improve knee cartilage thickness and volume in the mouse model. To perform a controlled experiment which utilizes a standardized jumping protocol for mice we designed and built the novel mouse jump (MJ) apparatus, since no such device was available in the market that allows for controlled jumping up or down. A group of four mice was hindlimb suspended (HLS) for nine weeks to generate and quantify any osteoarthritic response (specifically focusing on cartilage thinning), as expected based on previous work^17–19^. During the same time frame (Fig. 1), we trained another group of five mice (JUMP) in the MJ apparatus. The mice in the JUMP group performed jump training three times a week for 8 weeks (weeks 1–8), following a predetermined exercise protocol. The first week (week 0) was used to allow the mice to acclimate in the jumping machine. A control group of four mice (CON) was left in their regular cages for the same amount of time. All mice were hosted in the same environment for the same duration, and were fed with the same food/water, in an effort to standardize the process for better comparison of any negative or positive effects of the different protocols (training vs. unloading). Bone volume and density, cartilage thickness, and histology were assessed in all three groups. The positive effects on both cartilage and bone structure we measured in the JUMP group will serve as a basis for further investigations. We propose that plyometric training should be further assessed as a potential countermeasure to preserve healthy knee joint cartilage in long duration spaceflight, and as a preventive measure for patients at risk of developing osteoarthritis.

## Results

### Mouse model for jumping experiment

All mice from the three groups (HLS, JUMP and CON) were harvested at the end of the 8-week jumping experiment plus 1 week training. A schematic of the experiment is shown in Fig.1. In total, 6 mice in the CON group, 5 in the JUMP group, and 4 in the HLS group were analyzed. One hindlimb was prepared for micro-CT scans, the contralateral side was used for histology (see Methods).

### Body mass

**Fig. 2:**
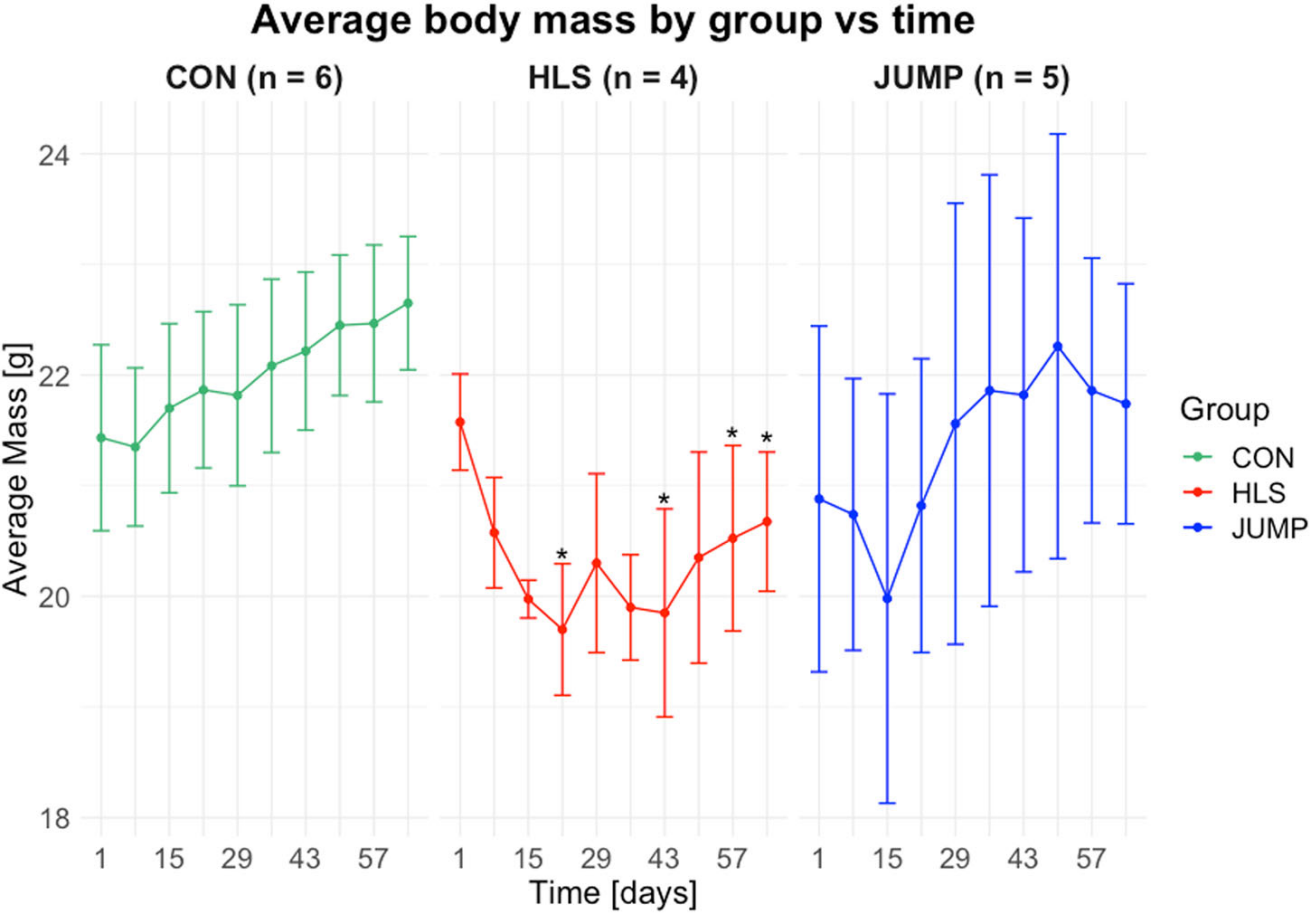
Average body mass per group during the experiment. Body mass was measured each week during the experiment. Data points represent the average measure for each group. Group size n is also reported for each group. Standard deviations are plotted as error bars. The measurements in the hindlimb suspended (HLS) group that are statistically different (*p* < 0.05) from control (CON) are marked with an asterisk.

Body mass was measured weekly for all the experimental groups (Fig. 2). At baseline, body mass was not different between groups. Both the HLS and JUMP groups showed immediate (likely stress-related) weight loss as compared to CON. The JUMP group recovered body mass within about 4 weeks since the beginning of the experiment. At the end of the experiment (day 64) the average body mass in the HLS group was lower than CON (HLS = 20.7 ± 0.6 g, CON = 22.6 ± 0.6 g, *p* = 0.012), while no difference can be established between JUMP (21.7 ± 1.1 g) and CON (*p* = 0.14).

### Micro-CT analysis reveals that jumping increases articular cartilage and bone thickness

We assessed cartilage thickness using contrast-enhanced micro-CT scans. 5% Phosphotungstic acid solution staining of the samples provided adequate contrast to visualize and measure the cartilage by micro-CT.

**Fig. 3:**
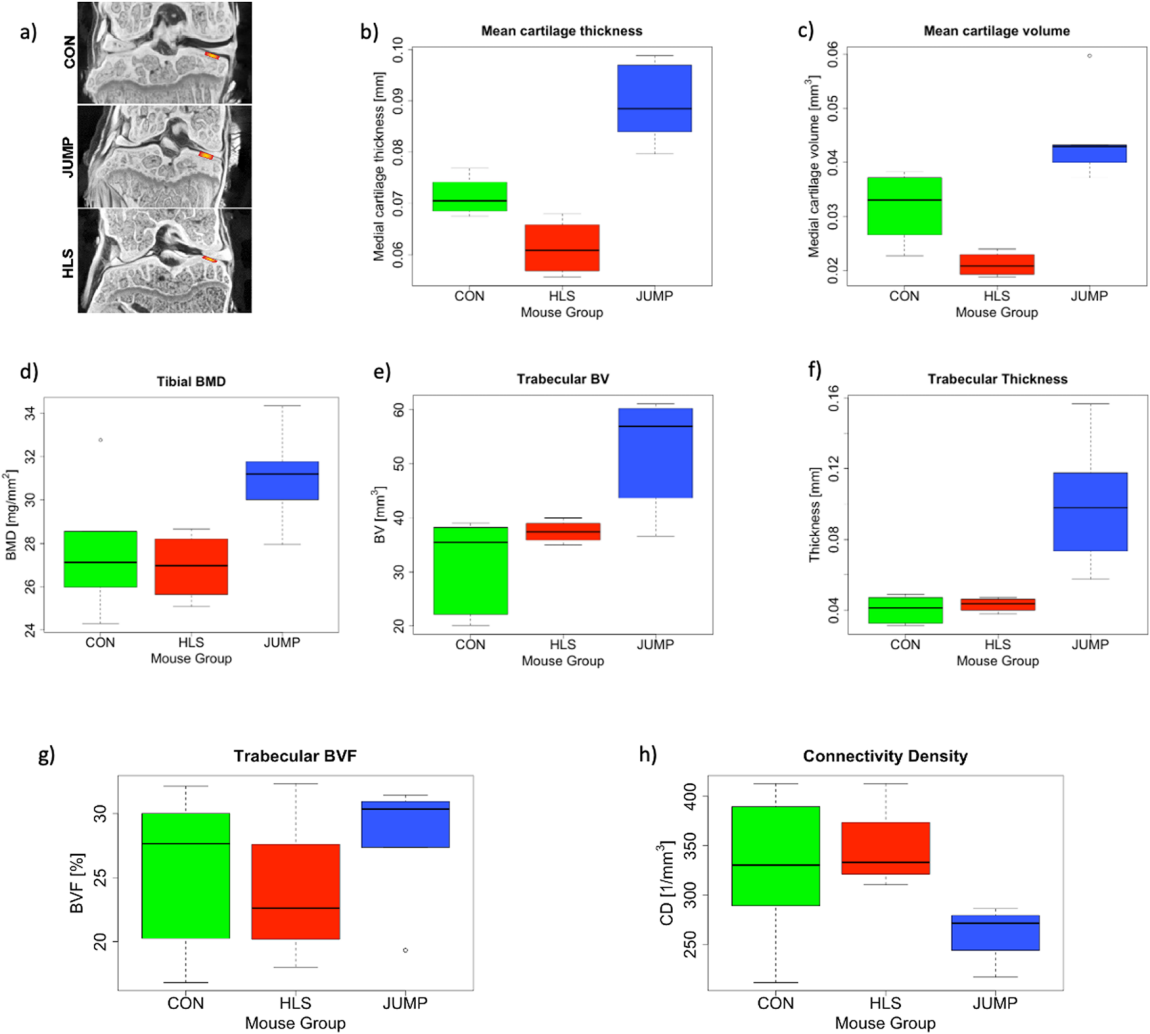
Effect of plyometric training on cartilage and bone structure by micro-CT analysis. **a** Representative contrast-enhanced micro-CT images of mouse tibia showing medial tibia-femur contact point (marked in red) where cartilage measurements were taken. **b**–**h** Boxplots for measurements of cartilage and bone structure parameters are shown in all other panels of the figure. Each of the groups are represented with a box of different colors and the corresponding group name is marked on the x axis of each plot. Control (green, CON, *n* = 6), jump (blue, JUMP, *n* = 5), hind limb suspended (red, HLS, *n* = 4). Each box indicates the interquartile range and whiskers indicate the range of the data, except for outliers which are marked with individual points, where present. Thick horizontal lines inside each box indicate median values for each group. **b** Mean cartilage thickness was measured in three groups. **c** Mean cartilage volume measured in all the groups in 200 μm tibial-femur contact point. **d** Tibial bone mineral density (BMD). **e** Quantification of trabecular bone volume (BV), **f** trabecular thickness, **g** Trabecular bone volume fraction, **h** Connectivity density.

Cartilage thickness at the medial tibial plateau was different (*p* = 4 × 10^–5^) between the three groups (Fig. 3, Table 1). After 9 weeks of hindlimb suspension, the mean cartilage thickness at the tibial-femoral cartilage-cartilage point of contact (Fig. 3a, b) is reduced by 14% in the HLS group with respect to CON (0.0613 ± 0.0056 mm for HLS vs 0.0713 ± 0.0037 mm for CON, *p* = 0.024) whereas the JUMP group shows a 26% mean thickening of the cartilage at the same location as compared to CON (0.0896 ± 0.0082 mm for JUMP, *p* = 0.0006). JUMP and HLS show the largest difference, as the cartilage thickness in JUMP is 46% higher than HLS (*p* = 4.2 × 10^–5^).

**Table 1:** Summary of the measured parameters and results of the pairwise t tests

Parameter (1)	CON (2)	HLS (3)	JUMP (4)	HLS vs CON (5)	JUMP vs CON (6)	JUMP vs HLS (7)
Cartilage thickness (mm)	0.0713 ± 0.0037	0.0613 ± 0.0056	0.0896 ± 0.0082	↓ 14% (*p* = 0.024)	↑ 26% (*p* = 0.0006)	↑ 46% (*p* = 4.2×10^-5)
Cartilage volume (mm³)	0.0318 ± 0.0061	0.0211 ± 0.0023	0.0446 ± 0.0088	↓ 33% (*p* = 0.026)	↑ 40% (*p* = 0.014)	↑ 110% (*p* = 0.0005)
Bone mineral density (BMD) (mg/mm²)	27.6 ± 2.93	26.9 ± 1.6	31.0 ± 2.3	Not significant (*p* = 0.659)	↑ 12% (*p* = 0.084) Not significant^*^	↑ 15% (*p* = 0.084) Not significant^*^
Trabecular bone volume (mm³)	31.7 ± 8.37	37.4 ± 2.1	51.7 ± 10.9	Not significant (*p* = 0.312)	↑ 63% (*p* = 0.052)	↑ 38% (*p* = 0.006)
Trabecular thickness (mm)	0.0404 ± 0.0074	0.0430 ± 0.0041	0.101 ± 0.039	Not significant (*p* = 0.861)	↑ 150% (*p* = 0.003)	↑ 135% (*p* = 0.006)
Trabecular bone volume fraction (BVF) (%)	25.8 ± 5.97	23.9 ± 6.05	27.9 ± 5.05	Not significant (*p* = 1)	Not significant (*p* = 1)	Not significant (*p* = 0.95)
Connectivity density (mm^–^³)	327.0 ± 73.2	347.0 ± 45.1	259.0 ± 28.8	Not significant (*p* = 0.58)	Not significant (*p* = 0.13)	Not significant (*p* = 0.10)

The parameters are listed in column (1), mean values and standard deviations for each group are reported in columns (2), (3), and (4). In column (5), (6) and (7) we report the results of the statistical tests quoting percentage differences between groups and *p*-values. Arrows up or down indicate an increase or a decrease in values.

NOTE: The results on BMD marked with an asterisk (*) are formally below our chosen level of statistical significance. However, for completeness, we include them in the table because the one-way analysis of means for BMD resulted in *p* = 0.05, i.e. at the threshold of statistical significance.

Similar results are observed when the overall cartilage volume of the medial tibial region is considered (Fig. 3c). Average cartilage volume is 33% lower in HLS compared with CON (0.0211 ± 0.0023 mm^3^ for HLS vs 0.0318 ± 0.0061 mm^3^ for CON, *p* = 0.026); medial tibial cartilage volume is 40% larger in the JUMP sample compared with CON (0.0446 ± 0.0088 mm^3^ for JUMP, *p* = 0.014). Volume in JUMP is 110% higher than in HLS (*p* = 0.0005).

Following this, the average value of tibial bone mineral density (BMD) is higher in the JUMP group by 15% as compared to the HLS group (31.0 ± 2.3 mg/mm^2^ for JUMP vs 26.9 ± 1.6 mg/mm^2^ for HLS, with low statistical significance, *p* = 0.084, Fig. 3d). Similarly, trabecular bone volume and trabecular thickness are higher in JUMP as compared to CON and HLS. For trabecular bone volume (Fig. 3e) the JUMP group is 38% (*p* = 0.006) and 63% (*p* = 0.052) higher than HLS and CON, respectively (51.7 ± 10.9 mm^3^ for JUMP, 37.4 ± 2.1 mm^3^ for HLS and 31.7 ± 8.37 mm^3^ for CON). For trabecular thickness (Fig. 3f), JUMP is higher than HLS by 135% (0.101 ± 0.039 mm for JUMP vs 0.0430 ± 0.0041 mm for HLS, *p* = 0.006) and higher than CON by 150% (0.0404 ± 0.0074 mm for CON, *p* = 0.003). These results indicate positive regulation of bone structure related to jumping mechanical loading. We also measured trabecular bone volume fraction and connectivity density (Fig. 3g, h). The measurements for these parameters do not show any statistical difference between the groups. All of these results are summarized in Table 1.

### Histological analysis shows clustering of chondrocytes in the HLS group

**Fig. 4:**
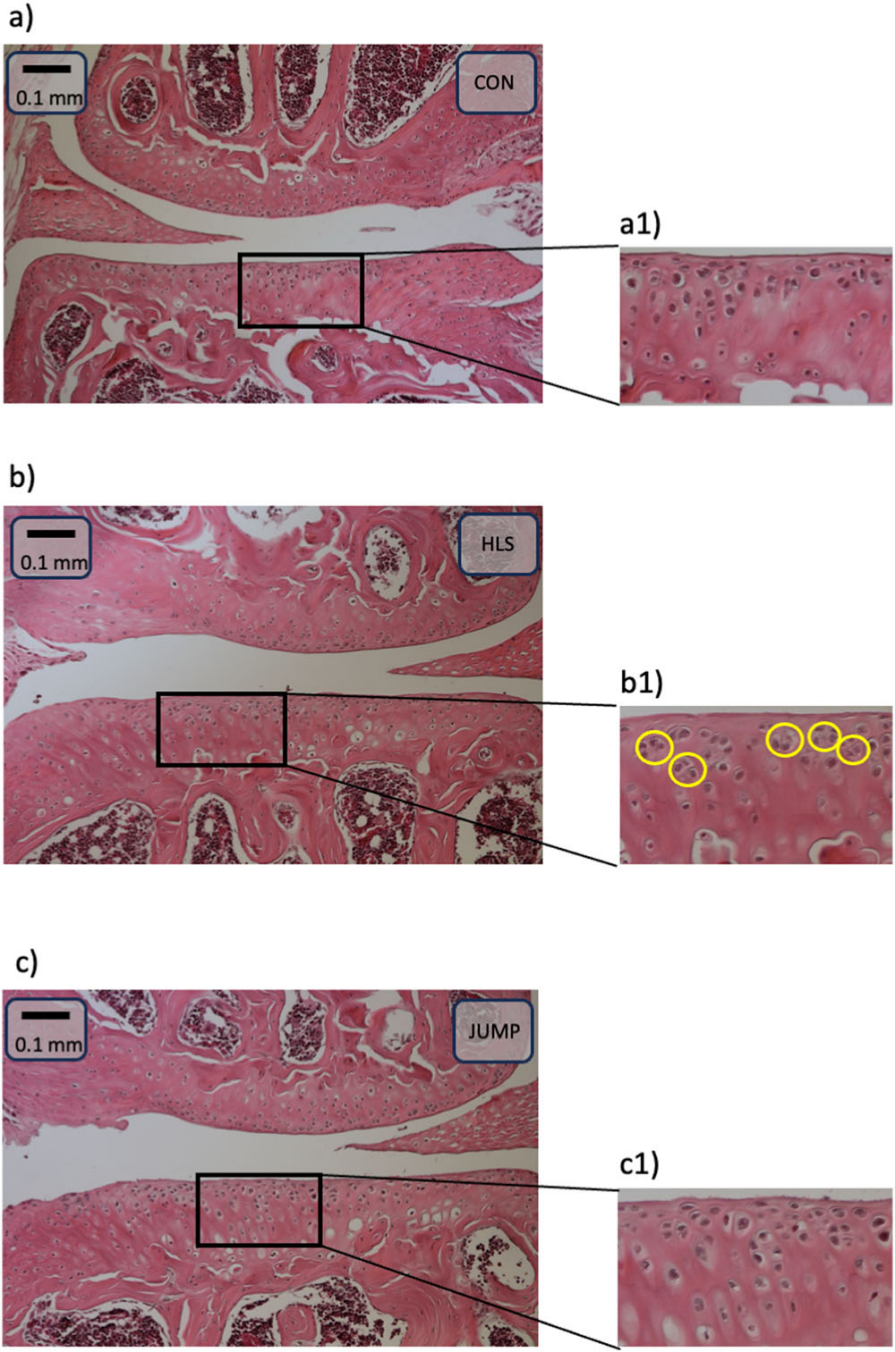
Histological images. Histological images of representative samples from each of the experimental groups are shown in the figure. The three large images show samples from the three groups: CON (**a**), HLS (**b**), JUMP (**c**). The scale bar is 100 microns, and the scale is the same in all three images. In the bottom right panel we show zoomed-in images to better show the cellular structure inside the black rectangles marked in each of the three groups (**a1** for CON, **b1** for HLS, **c1** for JUMP). Examples of clusters of chondrocytes, mostly present in the HLS group, are marked with yellow circles in panel **b1**. The size of the zoomed-in images is 0.25 mm × 0.15 mm.

Clusters of chondrocytes, defined as groups of cells that are separated by little extracellular matrix as compared to normal cells^16,26^, are visible specifically in the HLS group (Fig. 4). Enhanced clustering of cells is known to be associated with cartilage damage and osteoarthritis^26,27^. Normal cellular structure (i. e. elongated cells in the superficial layer and larger cells radially organized in the deeper zone^28^) is observed in the CON and JUMP groups.

We assessed the severity of osteoarthritis on the medial tibial plateau in each section utilizing the Articular Cartilage Structure (ACS) score for osteoarthritis^29,30^. No statistically significant differences were observed between the groups. The JUMP group exhibited a mean ACS score of 1.51 ± 1.64, while HLS had a mean ACS score of 1.64 ± 1.84 and the CON group had a mean score of 1.21 ± 1.54.

## Discussion

Healthy articular cartilage is a prerequisite for physical function. When symptoms of osteoarthritis manifest, activities of daily living may become extremely problematic and additional mechanical loading may pose an injury risk. There is growing evidence that long periods of immobilization, bed rest or exposure to microgravity may generate structural and molecular changes in the articular cartilage of synovial joints, leading to cartilage atrophy. Additionally, high radiation environments likely further contribute to cartilage damage^16,17^.

A key point of the experiment was to test the effects of unloading and jumping in a controlled environment, in order to minimize any external factors. The purpose of this study was twofold. 1) To measure the damaging effects of reduced loading in mice over a period of two months, using our own suspension cages. In line with previously published results, we expected to observe osteoarthritis phenotypic responses in the knee of a murine model, induced by reduced loading via hindlimb suspension. 2) To test the effects of plyometric training with our newly designed semi-automatic mouse jumping apparatus on specific properties of both knee cartilage and bone. While the beneficial effects of jumping have been documented for bones^31–33^, here we show the positive effects on joint cartilage. This information is essential to establish the pros and cons of plyometric training for preserving cartilage health, as well as evaluating the feasibility of the newly designed apparatus and the conditions under which we utilized it.

Plyometric training is a mode of exercise that enables a muscle to reach maximal force in the shortest possible time. An example of plyometric training is a “box jump”, in which the athlete quickly lowers his body by flexing the knees and the hips and then jumps to the top of a box with explosive power. Plyometrics is known to stimulate hormonal response, in particular growth hormone and IGF-1^24,34^. IGF-1 promotes cartilage repair by increasing cartilage anabolism^35^. There is also evidence from studies using bovine patellofemoral cartilage that dynamic compression increases protein synthesis^23,36,37^. Furthermore, when cyclic compression is applied in combination with IGF-I, this effect is significantly larger than either stimulus alone^25^. In various animal studies^38–42^, articular cartilage increased in thickness by up to 19–23%^41^ when high mechanical loads were applied. Series of repeated plyometric jumps can be used to generate cyclic compressive forces that might be beneficial to cartilage. However, it is important to consider that excessive forces may lead to cartilage damage. Interestingly, a positive effect of jump training on the biochemical properties of the patellar cartilage has been observed in a small human study^43^. Plyometric training was previously studied in the murine model only with respect to bone hypertrophy, showing that an 8-week protocol including 5 jumps/day (or more) significantly increases both tibial and femoral bone thickness^31^. Our experiment was thus aimed at directly comparing any positive or negative effects of either unloading or jump training on the mice knee cartilage.

In designing the experiment we considered the risk of overloading the joints. As for all modes of exercise, excessive joint loading (and training frequency) may lead to injury, possibly affecting cartilage structure^11–13^. Box jumps vs. drop jumps (i.e. jumping up vs jumping down) is a common example, where the latter should be used with extreme care to reduce risk of injury. Excessive eccentric forces upon landing may lead to muscle injury and ground reaction forces up to 7 times the body weight in human studies^44,45^. However, the exact volume/frequency that may lead to joint structural damage is not known, and should be further investigated in both humans and in the murine model. In our study, to minimize risk of joint damage in the mouse knee, we opted for a box jump (jump-up) type of exercise only. During the experiment, the mice jumped up from a lower to a higher platform, while we progressively increased the jump height and number of jumps during the course of the training period. This was aimed at progressively increasing the induced stimulus, and promoting adaptations, following the progressive overload technique^46^. Here we show that our approach led to positive results.

The damaging effect of hindlimb suspension is clearly observed in our HLS group compared with CON: we detected thinning of the tibial cartilage layer and smaller cartilage volume around the medial femoral-tibial cartilage-cartilage point of contact. The effect is still present when the entire medial tibial cartilage volume is considered. Our results are in line with the cartilage reduction measured in previous work, both in ground-based hindlimb suspension and rodent flight experiments on the ISS^17,19^.

The JUMP sample showed cartilage thickening and greater cartilage volume at the same location in the mouse knee compared to both CON and HLS groups. The difference observed between JUMP and HLS is not surprising, since the HLS group is negatively affected by reduced loading. However, the fact that both cartilage thickness and volume are higher in the JUMP group compared to the CON group is interesting in two ways: 1) the specific protocol we utilized did not cause any damage to the cartilage layer; and 2) the exercise protocol based on plyometric training produced a positive adaptation, increasing cartilage thickness and volume of the medial tibial plateau.

Similarly, the effect of jumping showed a positive impact on mouse bone. It is well known that high-impact mechanical loading like jumping exercises is beneficial to increase bone mass^47,48^. Here we observe a parallel trend in the JUMP mice group where there was an increase in skeletal features like tibial bone density, trabecular bone volume and thickness compared to the control. On the other hand, the HLS group in the present study showed decreased bone density and trabecular values compared to JUMP with no statistically significant difference when compared to the control group. Training like jumping during a period of disuse has been shown to maintain bone quality in young rats^32,33^. The intriguing possibility is that plyometric training may lead to a similar trend to preserve cartilage health during a period of disuse. This aspect should be explored in future investigations.

The histological analysis shows that clusters of chondrocytes are present in the HLS group, possibly indicating cartilage degradation due to unloading. The cell structure looks normal in the JUMP group, as compared to CON. Additionally, the ACS grading for osteoarthritis indicates no significant cartilage damage in the JUMP group (i.e., a low ACS score), which is consistent with the micro-CT measurements. This confirms that the mice in the JUMP group performing plyometric training maintained a healthy cartilage, and training did not cause any apparent change (i.e. damage) to the chondrocyte distribution in the extracellular matrix.

In summary, we showed that 1) reduced loading due to hindlimb suspension for 9 weeks caused cartilage thinning and clustering of chondrocytes, which have been observed as part of the arthritis responses; and 2) mice subject to 9 weeks of progressive plyometric training for 3 times a week showed increased cartilage thickness and volume while maintaining normal cellular structure. Further investigations should be performed to determine the best volume and frequency of training.

The observed cartilage thickening following a period of jump training is reminiscent of the results of previous experiments involving cyclic compression of cartilage explants^23^, and animal studies^41^ that showed increased cartilage thickness in the knee of the canine model after moderate running. While the details are not fully understood, low frequency (~0.1 Hz, or lower) dynamic compression appears to stimulate chondrocyte biosynthesis, possibly as a result of enhanced interstitial fluid flow^23^. IGF-1 produced during the jumping activity may also contribute, as the dynamic compression provided by the repeated jumps may increase transport of such a hormone to the articular cartilage extracellular matrix^25^. Our results corroborate these findings in the context of a specific exercise routine.

Whether the positive effects of plyometrics on cartilage health observed in our experiment with the mouse model can be transferred to humans is unknown. A further complication resides in the fact that adaptation of connective tissue during space flight or long-term bedrest is largely unknown. The possible application of plyometric training as a countermeasure against disuse induced changes should be carefully investigated from a multi-tissue perspective. It should also be kept in mind that the musculoskeletal system will deteriorate during spaceflight, which will affect tissue function. Nevertheless, we propose that plyometric training could be considered as an exercise mode for astronauts before flight, in anticipation of any negative effects due to microgravity during the mission. Plyometric exercise both as a strengthening routine and as a countermeasure to cartilage degradation during space flight could also be considered. In principle this can be achieved by designing specific machines. Since the two-month duration of the experiment with the mouse model corresponds to a time frame of about 4-5 years for humans, our results are likely relevant to long duration human space flight, such as a flight to Mars and return, or a tour of duty on the future lunar Artemis base. Furthermore, our results could potentially be important for prevention and/or rehabilitation protocols for osteoarthritis patients, or for the population at risk of developing such a disease.

## Methods

### The mouse jumping apparatus

**Fig. 5:**
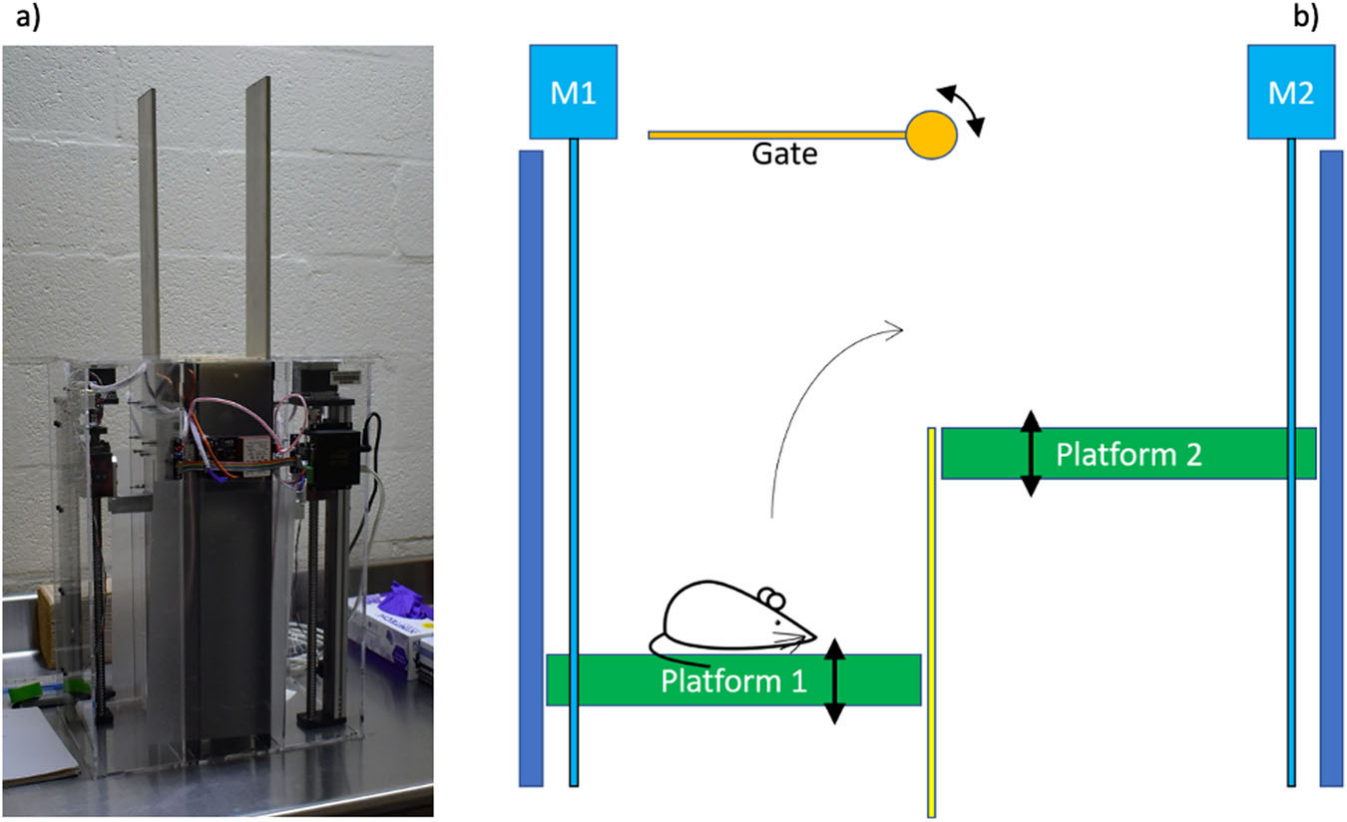
The MJ apparatus for mice plyometric training. **a** Full view of the MJ apparatus imaged on the lab table during the experiment. In panel **b** we show the basic mechanism of the apparatus. A mouse is sitting on the lower platform (1) when a green LED light (not shown) turns on. Shortly after that the gate opens and a mild electric shock is applied to the platform. The mouse learns that as soon as the gate opens she should jump onto the higher platform 2. When the mouse reaches platform 2, the gate closes, and the two platforms move (using motors M1 and M2) so that platform 1 is raised and platform 2 is lowered, and the cycle continues for the next jump, until the desired number of jumps is achieved. The platforms are located in the central (dark) part of the machine shown in panel **a**.

**Fig. 6:**
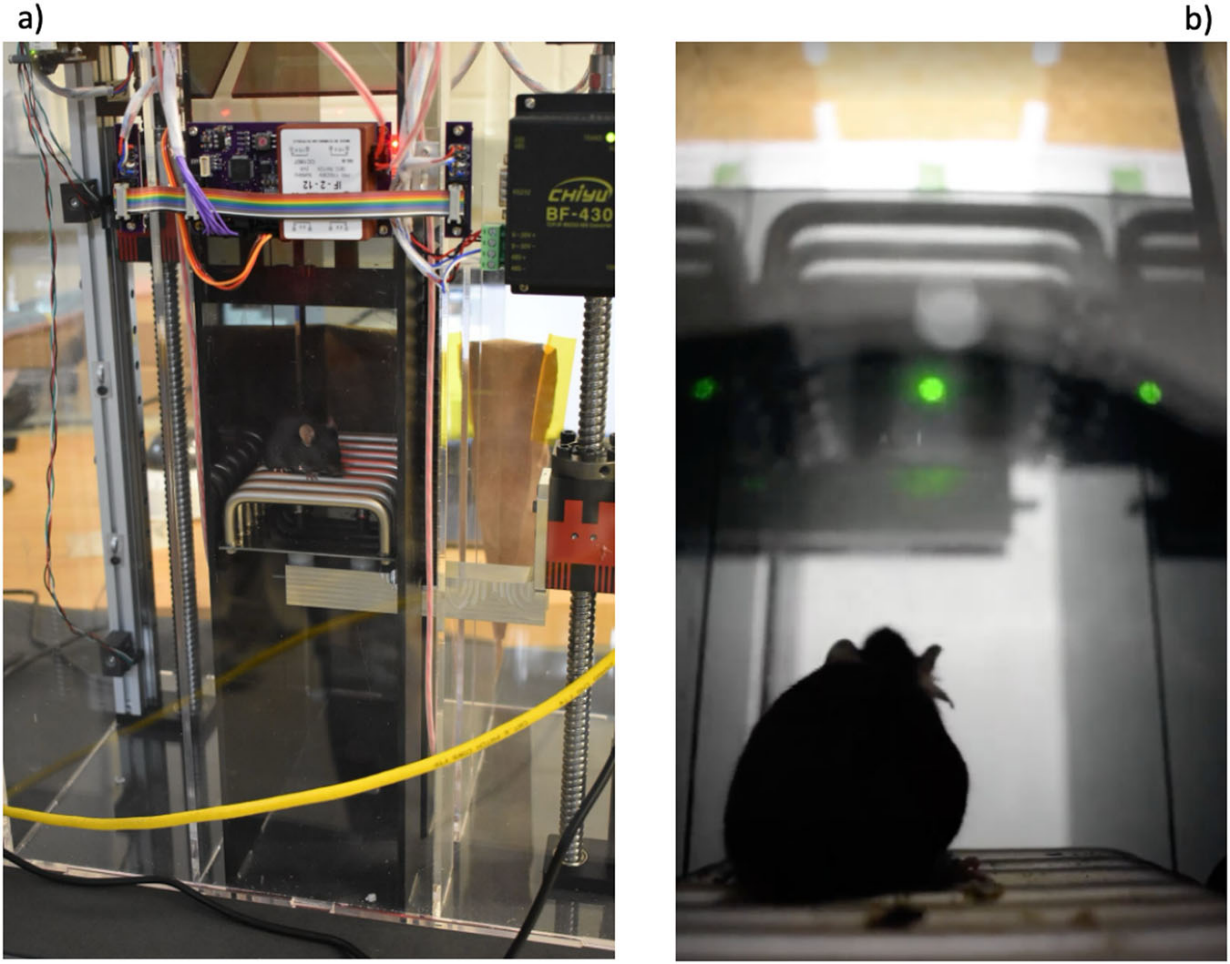
Close-up views of the MJ apparatus during standard operations. **a** A mouse is sitting on the lower platform as the platform is being moved down by the motor (red box on the right). The upper platform is located on the opposite side of the apparatus (not visible in this figure). **b** A mouse is sitting on the lower platform as the green LED turns on to signal the incoming shock. The gate to the upper platform is open and the mouse can jump up to the upper platform (visible above the green LED level). See videos of a jump in the Supplementary Videos SV1 and SV2 showing views from the bottom and the top, respectively.

We have designed and constructed the MJ (Mouse Jump) apparatus; a plyometric training device for one mouse that induces box jump motion using a green flashing light emitting diode (LED) followed by a 175 V_RMS_, 60 Hz AC voltage on the platform as the jump trigger. The device (Fig. 5a) has two movable platforms and the mouse is placed on one platform at the beginning of the cycle. Controlled by a custom Python algorithm, the platform hosting the mouse at the start of the cycle is lowered, while the second platform is raised to the desired height. As the platform stops, the green LED is activated to signal the incoming electric shock. The motorized gate opens prior to the jump and closes when the jump is complete (Fig. 5b). An infrared transmitter paired with a photodiode receiver on each platform senses whether the mouse is present on the elevated platform after the jump, and triggers the gate closure. Subsequently, the platforms are inverted so that the higher platform (which now hosts the mouse) is lowered and the mouse can repeat the jumps for the chosen number of repetitions. In Fig. 6 we show close-up views of the MJ apparatus. In Fig. 6a the mouse is sitting on the lower platform, ready to jump as soon as the green LED is activated and the gate is open. Figure 6b shows a view from the lower platform level, at the time when the green light is on and the gate is open to allow the jump to the upper platform. Supplementary videos SV1 and SV2 show a mouse jumping during the experiment, as seen from the bottom and from the top, respectively.

In the engineering phase of the project we tested MJ using slowly increasing voltages, making sure that the mouse would feel only a very mild electric shock. At the beginning of the experiment, we observed that the current is only relevant in the first training phase for each mouse, since the animal quickly learns to jump as soon as they see the LED light turning on and/or the gate opening.

The platform elevation is variable, with the elevation of each platform being controlled independently by a stepper motor driven linear stage. This control allows jump height to be changed via software over a range of 0–300 mm, and allows to carry out standardized, well controlled exercise regimens. The MJ software platform control can be set up to allow for jumps from lower to higher elevations or from higher to lower elevations. For this experiment, we only focused on jumping up to reduce the risk of overloading. Jump data is recorded by software. Collected data include the number of successful jumps, the jump height for each jump, and the number of failed jumps. A failed jump is defined as an event in which the mouse does not reach the upper platform before the hatch is closed. We observed a few of those events at the beginning of the training week, but none were recorded during the 8-week experiment.

### Mice

Animal treatment and care followed the rules and regulation of OLAW and the requirements of Carnegie Institution (vertebrate animal assurance # A3861-01). Procedures and husbandry of mice used in this project are described in animal protocol #163 and approved by Carnegie Institutional Animal Care and Use Committee (IACUC). The following descriptions of animal use are organized according to the ARRIVE Essential 10 guidelines 2.0. The study design was to test whether jumping exercise (JUMP) helps improve knee cartilage health. The jumping mice were compared to control (sedentary) and hind limb suspended (HLS) mice (also specified in the main text). The experimental unit is each mouse. C57BL/6J female mice were purchased from the Jackson Laboratory (JAX) at 2 months (mon) of age, acclimated to the new housing environment for one month, and used at 3 mon old. Total of 15 mice were assigned to 6 control mice, 5 JUMP mice and 4 HLS mice (see Experimental design section below for details). The sample sizes were determined based on prior published studies. The JUMP protocol is detailed in the Plyometric training protocol section below. Due to their same genetic background and sex, no specific criteria was applied to their exclusion. As such, data collected from all animals were included in tables and figures, including the n number for each group. Mice assigned to each group were chosen at random after receiving the shipment from JAX. To minimize environmental counforders, all mice were housed in the same room, with the same food, water, and bedding. Their health was monitored every other day (see blow). Due to experimental design, data recording was not blinded. However, at least two people had independently examined and analyzed the data for conclusions. Outcome measures included Micro-CT, histology, and body weight (details in relevant sections below and in the main text). The primary outcome measure was the articular cartilage health (thickness and OA score). Statistical methods and presentations are described below in the Statistical analyses section, as well as in figures and figure legends. The choice of pure bred C57BL/6J female mice of the same age (3 mon) was to ensure proper comparison among groups, and also because of their widespread use in research related to human health. Details for specific experimental procedures are described in several sections below. The results are reported in the main text and figures with means, standard deviations, and *p* values.

### Hindlimb suspension

We designed and constructed hindlimb suspension (HLS) cages to host 4 mice. Mice in the HLS group underwent a minor surgical procedure to implant a wire loop near the base of the tail for suspension, following the procedure described in ref. ^49^, similar to the NASA recommended procedure^50^ using the rat model. Briefly, one week before the HLS group reached 3 months of age, they were anesthetized by Avertin (Sigma; at 0.025%, 10 μl per gram body weight, via intraperitoneal injection) to reduce pain. Their tails then were cleaned with 70% ethanol before implanting 2-0 surgical steel sutures (Ethicon) through tail vertebrae. Extra-lengths of steel sutures were braided into extension loops (for suspension clips) above surgical sites, and surgical tapes were wrapped around the tails after being sprayed with Bactine, which is an antiseptic and pain reliever. After one week of recovery, the mice were subjected to HLS leading to unloading of the hind limbs. The steel loop on the tail was hooked to the clip at the end of an adjustable threaded plastic rod, which is attached to a horizontal PVC bar. The bar has rollers along the railway at the top of our custom-designed mouse tail suspension cage. For each mouse, the hindlimbs were lifted off the cage bottom at ~30° angle by adjusting the length of the plastic rod.

Each cage had two chambers (25 cm (W) × 25 cm (L) × 30 cm (H) each) divided by a clear acrylic divider with small holes that allowed nose-touch and social interaction between 2 animals. Water bottle, food tray, and woodchip bedding were placed inside each chamber and changed weekly.

The health status of the mice was monitored throughout the experiment. No signs of paralysis based on the movement of suspended hindlimbs and non-suspended forelimbs were seen. The urine color remained yellowish throughout (i.e. no accumulation of reddish porphyrin). There was likely stress caused by suspension, based on animals’ immediate weight loss and lagged recovery compared to other groups.

### Experimental design

A group of 4 mice were hindlimb suspended (HLS group) using custom-made suspension cages. A group of 5 mice were trained with jumps (JUMP group) three times a week, for a total of nine weeks, including one week at the beginning in which the mice got accustomed to the MJ machine. The jumping exercise protocol utilizes the standard progressive overload method to increase the stimulus during the program. We define the volume V of a training session as the height of the jump (i.e. the vertical distance between the two platforms in the MJ machine, h) and the number of jumps N for each mouse, in each session. The volume V = h * N is smaller at the beginning, and it is progressively increased every other week. More details on the protocol are given below in the appropriate section. After each training session, the JUMP mice were put back in their regular cages. A control group of six mice (CON) was housed in their regular cages for the total time of the experiment (9 weeks). All mice were in the same room at all times. No changes in the activity of the CON and HLS groups were made during the nine week duration of the experiment. Figure 1 shows a schematic of these 3 groups.

### Plyometric training protocol

Mice in the JUMP group exercised in the MJ apparatus three times a week (Mondays, Wednesdays and Fridays) for a total of nine weeks. The first week (Week 0) was dedicated to teaching the mice in the MJ apparatus, starting with a jump height of 5 cm. After 2–5 trials the mice learned how to avoid the electrical shock and jump to the elevated platform when the light flashed. When the mouse jumped confidently, we increased the jump height by 2 cm each time, until we reached a height difference of 15 cm between the platforms. This procedure was performed over four consecutive days, cycling through all of the five mice in the JUMP sample, until the mice confidently jumped at least 15 cm for at least 5 consecutive times. During the training week, the total number of jumps performed by each mouse was 23–41. In between each training session, the JUMP mice were housed in their regular cages.

**Fig. 7:**
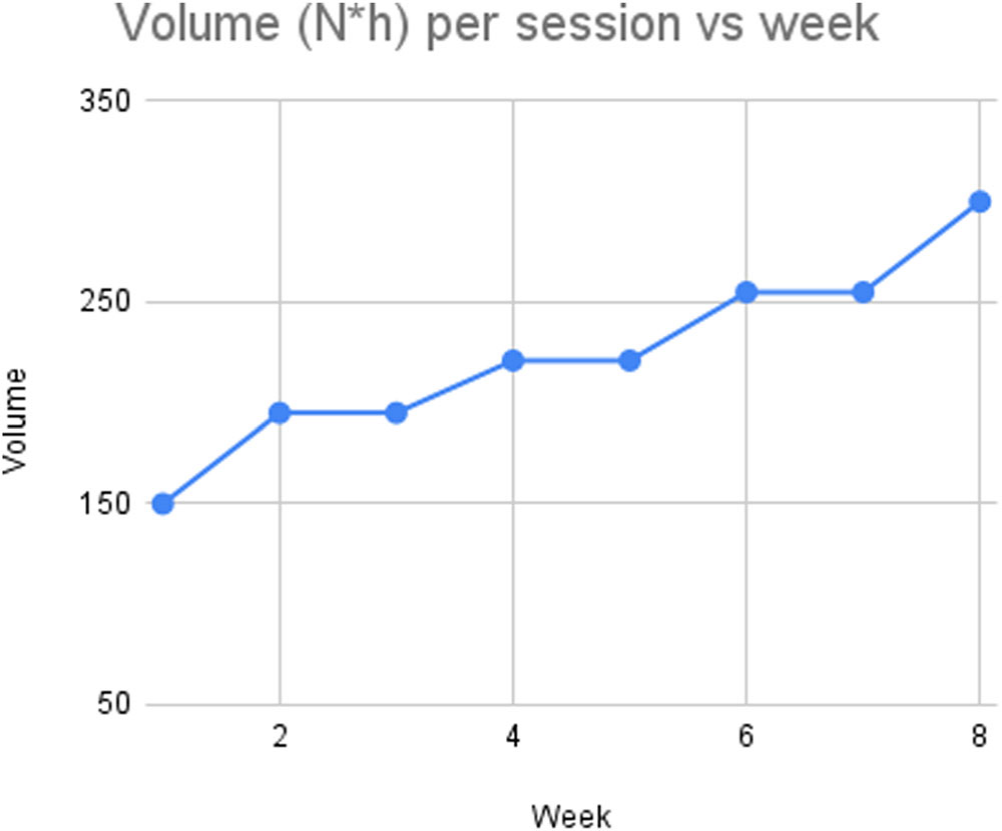
Volume of the training protocol per week. The plot shows the progressive change in exercise volume plotted against the week, from week 1 to 8 (end of the experiment). The volume is calculated as the number of jumps per session (i.e. each training day) multiplied by jump height in cm.

The exercise program started in week 1 and all JUMP mice followed a predefined progressive overload protocol. Progressive overload is a standard procedure implemented in human exercise programs for the purpose of gradually increasing the neuromuscular demand to facilitate further adaptations over time^46^. Exercise volume (V) per session was: V = N * h, where N is the number of jumps per session and h is the height (in cm). During the first week each of the mice performed 10 jumps with h = 15 cm, for a volume of 150. In Fig. 7 we show the progressive volume per session in each week, up to a value of V = 300 on week 8 (15 jumps, h = 20). Jump frequency depends on the time the platforms take to reach the predetermined height, and it was in the range ~0.01–0.05 Hz.

Jumping form and efficiency did not decrease over time, which could have signaled muscle-level damage. The mice quickly learned to jump over the first week of training and progressively improved their ability to reach greater heights and performed more jumps per session, as the protocol progressed to higher volumes.

### Tissue harvesting

Control, JUMP, and HLS groups were harvested on the same day at the end of the training period. Mice were anesthetized by Avertin (Sigma; at 0.025%, 10 μl per gram body weight, via intraperitoneal injection, prior to being sacrificed by cervical dislocation. The two-step euthanasia was used to reduce stress and pain. Afterwards, one hindlimb was dissected out and used for histological analysis, and the contralateral hindlimb prepared for micro-CT. Given there is no laterality of limb usage in rodents, left and right hind limbs were randomly chosen for either histological analysis or micro-CT.

### Histology

For histology, each removed hindlimb was immersed in 10% neutral buffered formalin (Polysciences) overnight, followed by decalcification in 10% EDTA/PBS (w/v) for 10 days, and dehydration and embedding in paraffin. Samples were sectioned at 5 μm thickness (frontal plane) and processed for hematoxylin-eosin staining per manufacturer’s (Surgipath) recommendations, and mounted in Micromount (Surgipath) with coverslip (VWR). At least 10 sections per mouse were examined. Images were taken using a Canon (EOS T3i) camera mounted on a Nikon E800 upright scope, under a 10X objective.

We assessed the severity of osteoarthritis on the medial tibial plateau in each section utilizing the Articular Cartilage Structure (ACS) score for osteoarthritis, which has been validated for use with hematoxylin-eosin staining^29,30^. We analyzed 10–14 mid-coronal sections from each group, with an average between 2 and 3 slices per mouse. Scoring was performed in a blind manner, by randomizing the images and concealing the group each image belongs to. Only after scoring all images the group was revealed. Scoring of three expert classifiers was then averaged. We tested for potential differences between the three groups as described in the Section Statistical Analysis.

### Contrast-enhanced Micro-Computed Tomography (micro-CT) and image analysis

To evaluate cartilage by micro-CT^19^, tibiae were fixed in 10% neutral buffered formalin for 3 days and further incubated in 5% Phosphotungstic acid solution at room temperature for 7 days. The samples were washed in PBS before scanning. The samples were imaged using an ex vivo high-resolution micro-CT imaging system (Bruker Micro-CT, Skyscan 1275, anode current 200μA; voltage 50 kV, use of 1 mm of an aluminum filter, scanning exposure time 218 ms with 0.3^o^ stem rotation, frame averaging of 6 with an image resolution of 5μm/pixel). After scanning, the image projections were reconstructed by the NRecom software (Bruker). CTVox and CTAan (Bruker) software were used for further analysis and visualization of reconstructed images. All images were oriented to align the knee-to-knee long axis in Dataviewer (Bruker). Tibial cartilage volume was measured at 200μm radii circular volume of interest of medial femur-tibial contact point within the cartilage (transaxial view of scans with a fixed number of slices for all samples). Tibial cartilage thickness was measured manually from the lower to upper margin of the cartilage coronal sections.

Bone-associated parameters (Tibial BMD, Trabecular BV, Trabecular thickness), to understand the effect on trabecular and cortical tibial bone were also assessed using the manufacturer software. For tibial cortical analysis, the mid-diaphyseal region was located using growth plate and notch after initial reorientation of all samples in the same position in Dataviewer software. Within the defined region of interest slices (ROI, approximately 60 slices)^51^, the cortical bone ROI is manually drawn including the outer periosteal surface. The cortical bone parameters were measured within a threshold of 90–255. For trabecular measurement, from the aligned scanned images, the growth plate and notch were noted initially. An offset of 25 slices from the growth plate was used and the analyses were performed between 150 and 200 slices for all the samples^51,52^. Next, the trabecular region of interest was manually drawn for all the slices of the samples. From the trabecular ROI and binary image verification, the bone parameters were measured with global thresholding between 110 and 255.

### Statistical analysis

Statistical analysis of the micro-CT measurements was performed using *R*^53^. The level of statistical significance was set at α = 0.05. Normal distribution was tested with the Shapiro-Wilk test (shapiro.test in *R*^53^) and homogeneity of variances with the LeveneTest (leveneTest^54^ in *R*). Both tests returned a *p*-value that does not allow for rejection of the null hypothesis, thus allowing the use of ANOVA (oneway.test in *R*^53^) to test for group (JUMP, HLS, CON) equality in cartilage and bone measurements. Additionally, we performed a pairwise post-hoc analysis to determine which group differs from each of the others using a Holm post-hoc method (pairwise.t.test in *R*^53^). All animal data were included for analyses., i.e. no exclusion criteria. The same statistical method was utilized to investigate differences in the ACS score for osteoarthritis and body weight between groups.

## Supplementary information

Supplementary Materials

Supplementary Video 1 (SV1). Mouse jump in the MJ apparatus (view from the lower platform level).

Supplementary Video 2 (SV2). Mouse jump in the MJ apparatus (view from the top of the apparatus).

## Data Availability

The datasets generated during and/or analyzed during the current study are available from the corresponding author on reasonable request.
