# Supplementary material for: Plyometric training increases thickness and volume of knee articular cartilage in mice
Source: NPJ Microgravity. 2025 Feb 13;11:5. doi: 10.1038/s41526-025-00458-z (PMC11825961; doi:10.1038/s41526-025-00458-z)
Supplement: Supplementary file 1 — Supplementary Materials [file 41526_2025_458_MOESM1_ESM.pdf]

Supplementary materials

Supplementary Video 1 (SV1)

Mouse jump in the MJ apparatus (view from the lower platform level).

In this video we show a mouse performing a jump during the experiment. The camera was placed just outside the MJ machine, at the level of the lower platform where the mouse was located before the jump. The gate that allows the mouse to jump onto the upper platform is already open at the beginning of the video. The green LED light signaling the incoming electric shock turns on after a few seconds and the mouse jumps to the upper platform before the shock is administered.

Supplementary Video 2 (SV2)

Mouse jump in the MJ apparatus (view from the top of the apparatus).

In this video we show a mouse performing a jump during the experiment. The camera was placed just outside the MJ machine, above the upper platform. At the beginning of the video the mouse is located on the lower platform (visible at the top of the image). The gate that allows the mouse to jump onto the upper platform (top of the image) opens after a few seconds in the video. The green LED light signaling the incoming electric shock turns on and the mouse jumps to the upper platform before the shock is administered.
